# The Continuous and Reversible Transformation of the Polymorphs of an MGAT2 Inhibitor (S-309309) from the Anhydrate to the Hydrate in Response to Relative Humidity

**DOI:** 10.3390/pharmaceutics16070949

**Published:** 2024-07-17

**Authors:** Tetsuya Miyano, Katsuji Sugita, Hiroshi Ueda

**Affiliations:** 1 Laboratory for Medicinal Chemistry Research, Shionogi & Co., Ltd., Osaka 561-0825, Japan; tetsuya.miyano@shionogi.co.jp (T.M.); katsuji.sugita@shionogi.co.jp (K.S.); 2 Analysis and Evaluation Laboratory, Shionogi & Co., Ltd., Osaka 561-0825, Japan

**Keywords:** crystal engineering, crystallization, hydrate, stability, polymorph

## Abstract

Polymorphic control is vital for the quality control of pharmaceutical crystals. Here, we investigated the relationship between the hydrate and anhydrate polymorphs of a monoacylglycerol acyltransferase 2 inhibitor (S-309309). Solvent evaporation and slurry conversion revealed two polymorphs, the hydrate and the solvate. The solvate was transformed into the hydrate by heating. X-ray powder diffraction demonstrated that the hydrate was transformed into an anhydrate via an intermediate state when heated. These crystal forms were confirmed under controlled humidity conditions; the presence of the anhydrate, the intermediate hydrate, or the hydrate depended on the relative humidity at 25 °C. The stoichiometry of S-309309 in water in the hydrate form was 4:1. The hydrates and anhydrates exhibited similar crystal structures and stability. The water of hydration in the intermediate hydrate was 0.1–0.15 mol according to the dynamic vapor sorption profile. The stability and dissolution profile of the anhydrate and hydrate showed no significant change due to similar crystal lattices and quick rehydration of the anhydrate. A mechanism for the reversible crystal transformation between the anhydrate and pseudo-polymorphs of the hydrate was discovered. We concluded that S-309309 causes a pseudo-polymorphic transformation; however, this is not a critical issue for pharmaceutical use.

## 1. Introduction

Crystallization is a critical process in the manufacturing of small-molecule pharmaceuticals. Drug molecules are regularly arranged in crystal lattices through weak but numerous intermolecular interactions such as van der Waals forces, hydrogen bonding, and electrostatic interactions [1]. Crystallization is commonly used in separation and purification processes. It involves the solidification of single molecules from solutions containing other compounds and depends on differences in their solubilities [2,3]. In manufacturing, scale-up is a vital factor for crystallization in which nucleation and crystal growth should be controlled [2]. The stability of the crystalline form of a drug molecule is also important. The quality control of drug products is a stringent requirement for the global manufacturing of pharmaceuticals; therefore, long-term stability and the detection and control of impurities and decomposition products are regulated [4,5]. Amorphous forms have favorable solubility and dissolution rates owing to their higher free energies than crystalline forms, but chemical and physical instabilities are often an issue for quality control [6,7]. The stability of a drug molecule can be enhanced by crystallization from the amorphous form, owing to the relatively low-energy state of the crystal lattice [1].

Polymorphs in the crystal form of pharmaceutical drugs are formed during the process of drug development. Most small-molecule drugs have at least two crystal forms (polymorphs) that arise from the formation of different crystal lattices through changes in the arrangement of drug molecules [8,9,10]. It is well known that the polymorphs of a drug have different physicochemical properties. These properties include solubility, the ability to form tablets, flowability, chemical and physical stability due to density, the state of intermolecular interactions, and the proportion of polar functional groups that cover the crystal surface [8,9,10,11]. Ritonavir is a useful illustration of the importance of pharmaceutical polymorphs. It was developed as an HIV protease inhibitor. However, it was withdrawn from the market owing to the generation of an unexpected polymorph with reduced solubility, which is critical for bioavailability and pharmacological activity [12]. This incident has significantly affected pharmaceutical research and the drug industry; consequently, there has been considerable focus on ritonavir polymorphs [13,14,15]. A new polymorph of ritonavir, Form III, was recently discovered [16,17]. Indomethacin, an ancient drug first marketed in 1965, is commonly used in pharmaceutical research. New polymorphs and their physicochemical properties have been reported in recent decades [18,19,20]. These cases imply that the comprehensive discovery and control of drug polymorphs prior to their release into the market is vitally important.

Multicomponent crystals comprising drug molecules and other molecules are of interest for crystal engineering. Salts are representative multicomponent crystals. The functional groups of the drug molecules ionize and form ionic interactions with organic or inorganic coformers. Approximately 40% of the first-in-class drugs approved by the United States Food and Drug Administration are salts, and numerous counterions are used as coformers [21]. The cocrystals have also emerged as salts. Non-ionic interactions play a role in the formation of crystal lattices between drug molecules and coformers. This enables the design of multicomponent crystals of drug substances without ionized functional groups [22]. Both salts and cocrystals can significantly affect physicochemical properties such as solubility and stability [21,22,23]. A multicomponent crystal is considered solvated if the coformer is liquid under ambient conditions. Organic solvents often form solvates with the drug molecules. However, this form should be treated carefully because residual solvents are strictly regulated for safety [24,25]. Water is a common and safe pharmaceutical coformer. Drug molecules form hydrates with water, and the number of such hydrates has significantly increased in recent decades [24]. Drug molecules can become hydrated or dehydrated depending on the ambient conditions [26]. This is an important consideration in crystal engineering.

Recently, the market for anti-obesity treatments has grown drastically due to the release of glucagon-like peptide 1 receptor (GLP-1R) agonists, which can help reduce body weight and showcases antidiabetic effects [27]. With increasing investment in the anti-obesity market, monoacylglycerol acyltransferase 2 (MGAT2) is considered a pharmacological target for anti-obesity treatments [28,29]. It is highly expressed in the small intestine and catalyzes triacylglycerol synthesis during absorption. The inhibition of MGAT2 can suppress food intake in mice fed a high-fat diet through peripheral vagus nerve signaling and has potential as a novel anti-obesity strategy [29]. In the present study, we aimed to identify and control the polymorphs of a drug originally designed for MGAT2 inhibition. First, we performed polymorphic screening and classified the crystal forms obtained. We determined the physicochemical properties and transformation mechanisms of the polymorphs.

## 2. Materials and Methods

### 2.1. Materials

S-309309 was originally designed as an MGAT2 inhibitor and synthesized by Shionogi & Co., Ltd. (Osaka, Japan) [30,31,32]. Ethanol (EtOH), methanol (MeOH), isopropanol (IPA), and ethyl acetate (AcOEt) were purchased from Fujifilm Wako Pure Chemical Corporation (Osaka, Japan). Purified water was obtained from Otsuka Pharmaceutical Co., Ltd. (Tokyo, Japan). The molecular weights and pKas were determined from the PhysChem Profiler Module using ACD/Percept ver. 14.3.0 (Advanced Chemistry Development, Toronto, ON, Canada).

### 2.2. Polymorphic Screening

#### 2.2.1. Solvent Evaporation

Approximately 10 mg of S-309309 was placed in an S-1 4 mL glass vial (Nichiden Rika Glass Co., Ltd., Hyogo, Japan), and each solvent (MeOH, EtOH, IPA, AcOEt, and water) was added. Solubilization was confirmed by observation. S-309309 was dissolved in 1 mL of AcOEt at ambient temperature (approximately 20–23 °C) and 1 mL of MeOH at 60 °C. EtOH, IPA, and water were unable to solubilize S-309309. The resulting solutions and suspensions were subjected to solvent evaporation at 30 °C and 1750 rpm under reduced pressure using a Genevac HT-8 Series II evaporation system (Genevac Ltd., Ipswich, UK), and the residual powders were obtained.

#### 2.2.2. Slurry Conversion

Two hundred micrograms of solvent and 10 mg of S-309309 were added to an S-1 4 mL glass vial (Nichiden Rika Glass Co., Ltd., Hyogo, Japan) equipped with a magnetic stirrer. After the cap was tightened, slurry conversion was performed at 300 rpm at ambient temperature using a CPS-300 cool stirrer (Scinics Corporation, Tokyo, Japan). After 8 days of slurry conversion, each suspension was treated by suction filtration using OmniporeÒ and a 0.45 mm polytetrafluoroethylene membrane (Merck KGaA, Darmstadt, Germany). The filtrate on the membrane was obtained.

### 2.3. X-ray Powder Diffraction

#### 2.3.1. X-ray Powder Diffraction

The crystal forms of the samples were determined by X-ray powder diffraction (XRD) using a D8 Discover (Bruker AXS K.K., Kanagawa, Japan) or SmartLab (Rigaku Corporation, Tokyo, Japan) system. The Cu Kα radiation point source was operated at 40 kV and 40/200 mA. Other procedures were performed as in previous reports [33,34]. The data were analyzed using GADDS for XP/2000 ver. 4.1.27 (Bruker AXS K.K., Kanagawa, Japan) or Smart Lab studio II X64 version 4.2.111.0 software (Rigaku Corporation, Tokyo, Japan).

#### 2.3.2. XRD under Controlled Temperature and Humidity

Changes in the crystal forms of the samples under temperature and humidity control were recorded using a Rigaku RINT 2100 Ultima instrument combined with XRD-DSC Thermo Plus II and HIM-1 systems (Rigaku Corporation, Tokyo, Japan). Approximately 2–3 mg of each sample was placed on an XRD/DSC aluminum pan (7 × 7 × 0.3 mm), and its XRD pattern was obtained while heating at a rate of 5 °C/min from room temperature (approximately 21–25 °C) to 80 °C. The effect of humidity was studied on the crystal form analyzed at 25 °C with relative humidity (RH) from 25% to 60% with 5% intervals. The voltage and current were set at 40 kV and 40 mA, respectively. The X-ray data were collected in the range of 5–35° (2-theta) at a scan rate of 60°/min with a scan step of 0.02°. The resulting data were analyzed using XRD-DSC ver. 2. 04 (Rigaku Corporation).

### 2.4. Thermal Analyses

#### 2.4.1. Differential Scanning Calorimetry

Differential scanning calorimetry (DSC) was performed to determine the heat flow profile of S-309309 using a Discovery Q-1000 system (TA Instruments Japan, Tokyo, Japan) with nitrogen as the purge gas, supplied at a rate of 50 mL/min and calibrated using an indium standard. Each sample (1–3 mg) was weighed in an aluminum pan, which was then sealed. The thermal behavior of each sample was investigated over the temperature range of 0–250 °C at a rate of 10 °C/min. Dehydration behavior and melting point (*Tm*) were determined using Universal Analysis 2000 ver. 4.7A (TA Instruments, Tokyo, Japan).

#### 2.4.2. Thermogravimetric–Differential Thermal Analysis

Thermogravimetric–differential thermal analysis (TG-DTA) was performed using a STA7200RV instrument (Hitachi High-Tech Science Corporation, Tokyo, Japan). Each sample (1–5 mg) was placed in an aluminum pan, and its change in weight and thermal profile were determined at 10 °C/min from room temperature (21–25 °C) to 300 °C. Data were analyzed using TA7000 standard analysis version 11.2 (Hitachi High-Tech Science Corporation, Tokyo, Japan).

### 2.5. Raman Spectroscopy

The Raman spectra of S-309309 were obtained using a Raman touch laser microscope (Nanophoton Corporation, Osaka, Japan). Each sample was placed on an aluminum plate, and its Raman spectrum was obtained using the following parameters: excitation wavelength, 671 nm; excitation power, 130.56 mW; ND filter, 56.31%; spectrograph center wavelength, 1400 cm^−1^; grating, 600 gr/mm; slit width, 50 μm; exposure time, 0.5 s; averaging, 10; gain, high; readout port, low noise; readout speed, 2 MHz; CCD temperature, −70 °C; objective lens, 5×/NA 0.15. The wavenumber was calibrated using the Si spectrum provided by the equipment. The peak positions in the Raman spectra were assigned using a smoothing process based on the fast Fourier transform method. The spectra were analyzed using a Raman Viewer (Nanophoton Corporation, Osaka, Japan).

### 2.6. Dynamic Vapor Sorption

The dynamic vapor sorption (DVS) profile of the water sorption and desorption of S-309309 was obtained using a DVS Advantage system (East Core Ltd., Tokyo, Japan). Each sample (8–9 mg) was weighed and placed in a pan. Water sorption was gravimetrically measured at 25 °C under various RH conditions. The RH was increased from 0% to 90% at a rate of 0.02%/min in 10% steps and was maintained until the weight change reached a plateau at each RH. The results were analyzed using the DVS Advantage control software ver. 2.1. 0.9 (East Core, Ltd., Tokyo, Japan).

### 2.7. Single-Crystal XRD and Structural Analysis

Single-crystal XRD data were collected using a Rigaku XtaLAB P200 system with CrysAlisPro 1.171.39.46e software (Rigaku Oxford Diffraction) using thin-layer mirror monochromated Cu-Kα radiation (λ = 1.54184 Å). The hydrate was mounted and examined at 213 K under a dry nitrogen purge and re-examined following transformation to an anhydrate at 298 K. The direct SHELXT method was used for the structural solution of the crystals [35]. All calculations were performed with the observed reflections [I > 2σ(I)] with CrysAlisPro 1.171.39.46e (Rigaku Oxford Diffraction), except for refinement, which was performed using the SHELXL program [36]. All nonhydrogen atoms were refined with anisotropic displacement parameters, and hydrogen atoms were placed in idealized positions and refined as rigid atoms with relative isotropic displacement parameters without hydrated water. Packing images and void spaces were prepared using Mercury.

### 2.8. Stability Test

The stability test was performed under accelerated conditions. The sample was stored in each desiccator with silica gel or saturated solution of sodium chloride (75% RH). The desiccators were stored at 40 °C for 2 months. The purity of the samples before and after storage were measured by using a high-performance liquid chromatography system Prominence (Shimadzu Corporation, Kyoto, Japan). The measurement conditions were as follows: mobile phase, 0.1% formic acid aqueous solution/acetonitrile with 0.6 mL/min; column, YMC-Triart C18 ExRS at 40 °C; injection volume, 3 μL; UV at 266 nm wavelength.

### 2.9. Dissolution Test

A dissolution test was performed using a μDiss with UV monitoring system (pION Inc., Billerica, MA, USA). Phosphate buffer (pH 6.8) was prepared as a simulated intestinal medium according to the Japanese Pharmacopoeia—18th edition, and 20 mL of the medium was placed in a dissolution vessel. Approximately 5 mg of the sample was weighed into a dissolution vessel, and the dissolution test started with stirring at 300 rpm at 37 °C. The drug concentration was monitored using a UV probe (5 mm) inserted into the donor at 240 nm every 1 min for 120 min.

## 3. Results and Discussion

### 3.1. Chemical Structure and Physicochemical Properties

**Figure 1:**
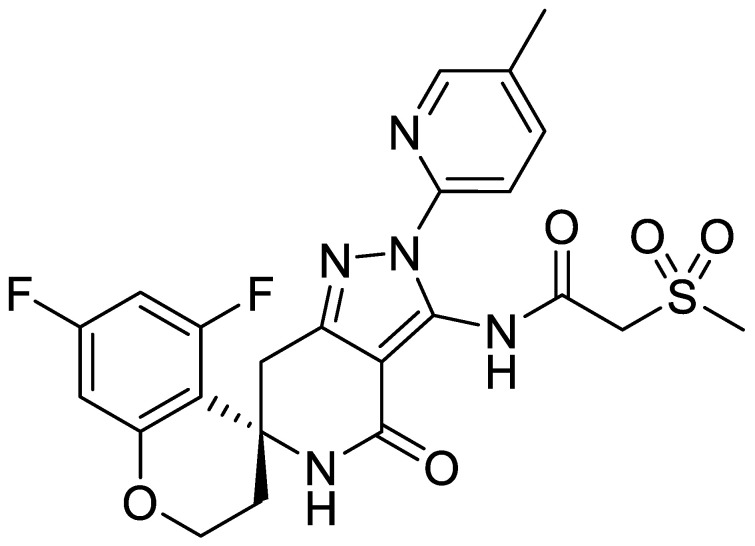
Chemical structure of S-309309.

The chemical structure of S-309309 is shown in Figure 1. Its physicochemical parameters are as follows: molecular weight, 517.51; log P, 1.92; topological polar surface area, 140.66; H-bond donors, 2; H-bond acceptors, 10; and pKa (acid/base), 10.5/2.3. Lipinski’s five rules (molecular weight < 500, log P < 5, topological polar surface area in the range of 0–140, H-bond donors < 5, and H-bond acceptors < 10) have traditionally been used as critical indicators of drug-like molecules [37]. The physicochemical parameters of S-309309 were within Lipinski’s five rules, suggesting a drug-like molecule, although the number of H-bond acceptors was not less than ten.

**Figure 2:**
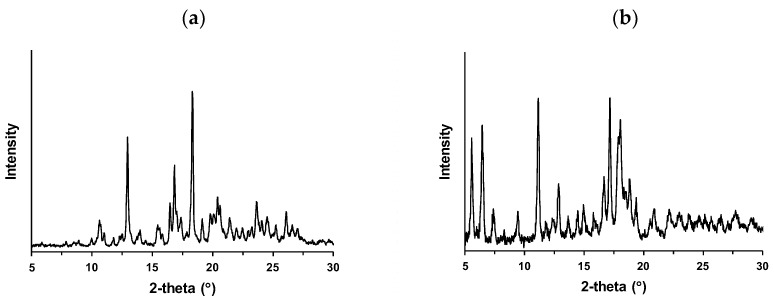
X-ray powder diffraction (XRD) profiles of (**a**) Form I and (**b**) Form II.

**Figure A1:**
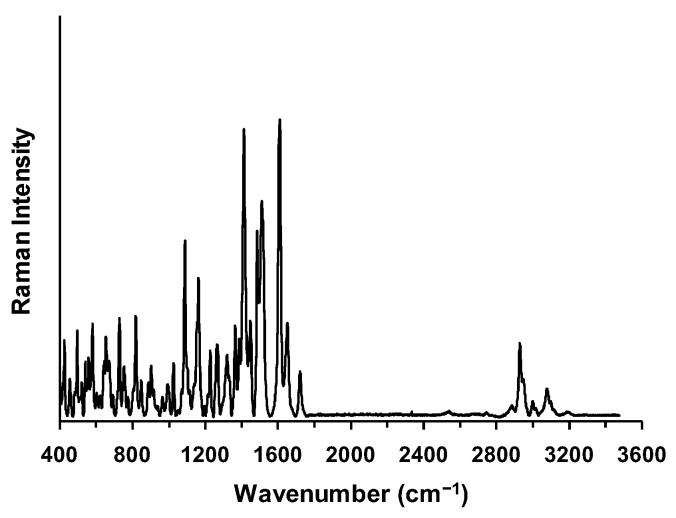
Raman spectrum of intact S-309309.

**Figure A2:**
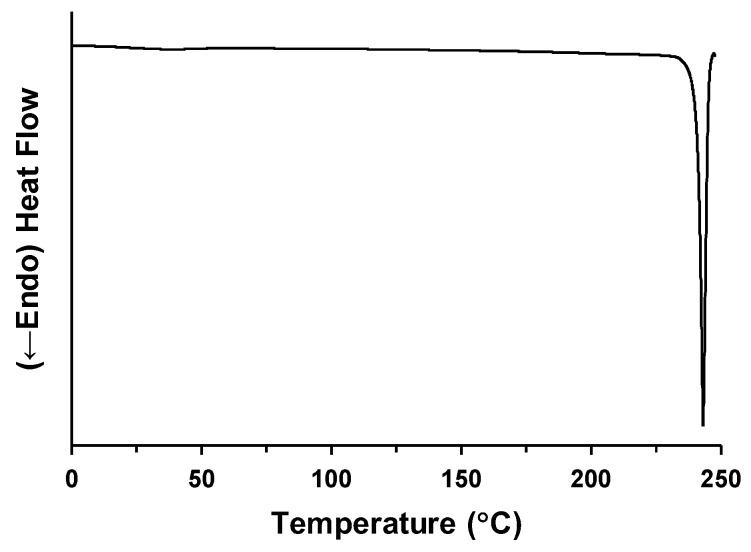
Differential scanning calorimetry (DSC) profile of intact S-309309.

Figure 2a shows the XRD profile of intact S-309309. Typical XRD peaks are shown (Table A1), suggesting the formation of the native crystal form of S-309309, designated as Form I. The Raman spectrum of intact S-309309 is shown in Figure A1. The Raman peaks were characterized especially in the fingerprint region at approximately 400–1900 cm^−1^ (Table A2) according to the vibrational and rotational changes of the incorporated functional groups [38]. Thermal behavior was investigated by DSC. Figure A2 shows the heat flow profile of intact S-309309. A small endothermic peak was observed at 30–50 °C; the onset and peak top were 11.0 and 34.4 °C, respectively. This thermal event suggests crystal transformation or desolvation/dehydration. The second endotherm shows a relatively large peak corresponding to the melting behavior, with the onset and peak top at 237.3 and 243.0 °C, respectively.

### 3.2. Polymorphic Screening

The crystal form of intact S-309309 (Form I) was investigated by solvent evaporation and slurry conversion because solvent-mediated crystal transformation is well known [8,9,10,11,12,13,14]. Table 1 summarizes the polymorphic screening results. None of the samples obtained by solvent evaporation exhibited crystal transformation and existed as Form I, except in the case of AcOEt. The sample obtained by the evaporation of AcOEt showed no XRD peaks corresponding to a halo pattern, suggesting amorphization. However, a new crystal form was observed after the slurry conversion. MeOH, EtOH, IPA, and water produced Form I after slurry conversion; however, EtOAc produced a different crystal form, classified as Form II. Figure 2b shows the XRD profile of Form II. As shown in Figure 2a and Table A1, the representative XRD peaks at 10.6°, 13.0°, 16.5°, 16.8°, 17.0°, and 18.3° and multiple peaks in the range of 19–22° were observed for Form I. Form II produced a different pattern from that of Form I, and the assigned XRD peaks of Form II are shown in Table A3. Peaks at 5.6°, 6.5°, 7.4°, and 9.4° appeared in the range of 5–10°, although Form I did not produce typical peaks. The peaks at 16.7° and 17.2° are strong; however, it is difficult to differentiate between the peaks of Form I. A doublet peak at 17.9°/18.0° was observed for Form II.

**Figure 3:**
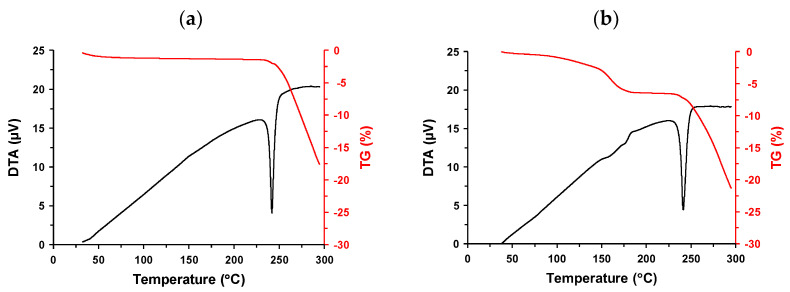
Thermogravimetric–differential thermal analysis (TG-DTA) profiles of (**a**) Form I and (**b**) Form II.

The thermal behavior and weight of both Forms I and II were determined as a function of temperature. Figure 3a,b show the TG-DTA profiles of Forms I and II, respectively. Form I exhibited a small weight loss corresponding to approximately 0.8% up to 100 °C where typical thermal events were not observed. Further heating included an endothermic peak at 235.6 °C (onset)/242.0 °C (peak top). Following melting, weight loss >15% was observed. A similar TG-DTA profile was observed for diclofenac salt [39]. The first small weight loss may be attributed to dehydration because Form I was obtained from various solvents (Table 1), suggesting a hydrated form. The endothermic peak represents a melting event, which almost agrees with the DSC measurements (Figure A2). The rapid reduction in weight over 245 °C reflects thermal degradation. There was relatively large weight loss (approximately 6.4%) in the TG-DTA profile of Form II until 195 °C. The DTA profile also featured weak but multiple endotherms in the range of 150–195 °C, suggesting desolvation/dehydration. This event can be considered as desolvation because Form II was obtained only from AcOEt, suggesting that it was an AcOEt solvate. There was a melting peak at 236.4 (onset)/241.3 °C (peak top), which was not significantly different from that of Form I. This suggests a crystal transformation to Form I after the desolvation of Form II. From a safety perspective, a solvate containing an organic solvent is undesirable as a pharmaceutical solid form from the perspective of safety [25]. Therefore, Form I was subjected to further investigations.

### 3.3. Dependence of Crystal Transformation on Temperature

**Figure 4:**
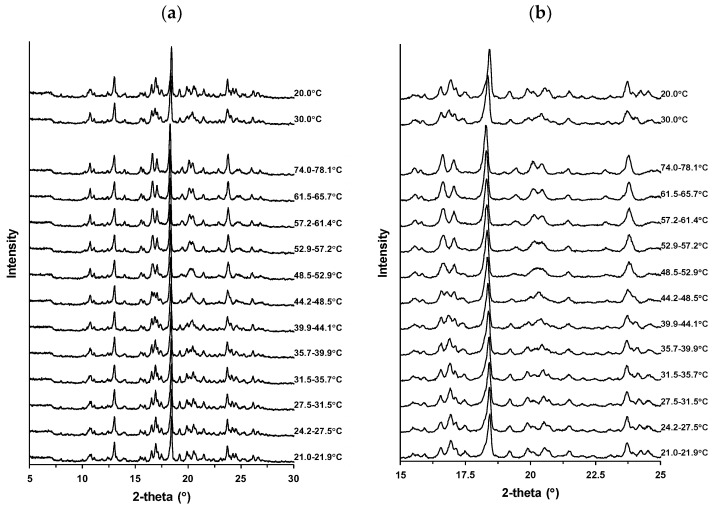
X-ray powder diffraction (XRD) profiles of Form I obtained at various temperatures: (**a**) overall (5–30°) and (**b**) magnified (15–25°).

XRD measurements were performed at controlled temperatures to investigate the thermal events indicated in the TG-DTA profile of Form I (Figure 3). Figure 4a shows the XRD profiles of Form I obtained while heating from approximately 20 to 80 °C and following cooling to 30 °C and 20 °C. The XRD profile obtained at 21.0–21.9 °C was similar to that shown in Figure 2. This profile was retained almost up to 35.7–39.9 °C. However, changes in the XRD peaks started from 39.9 to 44.1 °C. The peak at 10.6° sharpened, and the peaks near 16–18°, 19.5–21°, and 23.5–25° changed in number, position, and shape depending on the temperature. The magnified profiles at 15–25° are shown in Figure 4b. These changes almost reached a plateau at 57.2–61.4 °C, suggesting crystal transformation. After heating to approximately 80 °C, the samples were cooled to ambient conditions and maintained at 30 °C. Further, the XRD profile reverted to the intact profile and almost recovered to that of the intact Form I before heating at 20 °C.

Crystal transformations during heating have also been reported for other drugs. For example, theophylline has pseudo-polymorphs between anhydrate and hydrate, and its dehydration behavior has been confirmed from an endotherm in its DSC profile and a change in the XRD profile after heating [40,41]. Prednisolone forms a sesquihydrate, and heating induces dehydration and the formation of a metastable anhydrate [42]. The dihydrate of ondansetron hydrochloride exhibited a change in its XRD profile, reflecting dehydration during heating, and formed an anhydrate via a hemihydrate as an intermediate state [43]. The XRD profiles of S-309309 (Form I) observed in the present study demonstrated a similar tendency under controlled temperatures. A small weight loss in Form I up to 100 °C was also apparent in the TG-DTA profile (Figure 3). The continuous change in the XRD profile of Form I reflects the dehydration of the stable anhydrate. Intermediate crystals of a metastable anhydrate and pseudo-polymorphic hydrate were generated at approximately 40–50 °C. The stable hydrate reversibly recovered to an intermediate state and an anhydrate at 30 and 20 °C, respectively.

### 3.4. Dependence of Crystal Transformation on Temperature and Humidity

**Figure 5:**
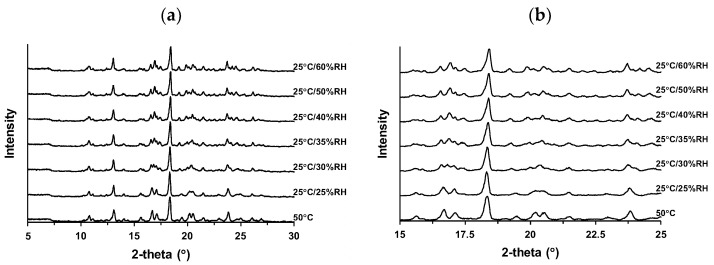
X-ray powder diffraction (XRD) profiles of Form I obtained at various temperatures and humidities: (**a**) overall (5–30°) and (**b**) magnified (15–25°).

The change in the crystal form of Form I (Figure 4) in response to RH was investigated to elucidate its intermediate state. Figure 5a shows the XRD profiles of Form I under controlled temperature and humidity conditions, and the magnified profile in the 15–25° range is displayed in Figure 5b. The XRD patterns are listed in Table A4. First, Form I was measured at 50 °C as the stable anhydrate according to the results shown in Figure 3 and Figure 4. The peaks at 15.6°, 16.7°, 17.1°, 18.3°, 19.5°, 20.2°, 20.5°, 21.5°, and 23.8° were typical. The XRD profile obtained at 25 °C/25% RH did not change significantly, suggesting the retention of stable anhydrates. However, there was a change in the XRD profile at 25 °C/30% RH, and a plateau was almost reached at 25 °C/35% RH. There were peaks at 15.5°, 16.6°, 16.9°, 17.4°, 18.4°, 19.3°, 20.0°, 20.4°, 21.4°, 23.7°, 24.1°, and 24.6°. This change in the XRD profile suggests the formation of an intermediate state. This crystal form was assumed to be an intermediate hydrate because its generation is dependent on humidity. Further humidification caused changes in this profile. The XRD profile obtained at 25 °C/60% RH reveals the growth of peaks at 16.9°, 23.7°, and 24.6°. Peaks at 20.0° and 20.4° formed doublets at 19.9°, 20.1°, 20.5°, and 20.7°, suggesting the formation of pseudo-polymorphic hydrates.

Reversible hydration and dehydration behaviors were confirmed for other drugs. Prazosin hydrochloride forms a nonstoichiometric polyhydrate in response to humidity, and its XRD and DVS profiles reveal reversible dehydration at relatively low humidity [44]. Paroxetine hydrochloride crystals show conversion between stoichiometric and non-stoichiometric hydrates depending on the humidity [45]. We hypothesized that Form I of S-309309 forms three different states: an anhydrate (Form I-A) at 25 °C/25% RH; an intermediate hydrate at 25 °C/35% RH (Form I-B); and a hydrate at 25 °C/60% RH (Form I-C) according to the temperature and humidity. Form I-A, Form I-B, and Form I-C almost corroborated with the XRD profiles at 21.0–21.9 °C, 35.7–39.9 °C, and 52.9–57.2 °C in Figure 4, respectively, suggesting that these crystal forms can be controlled by regulating only the temperature.

### 3.5. Water Sorption and Desorption

**Figure 6:**
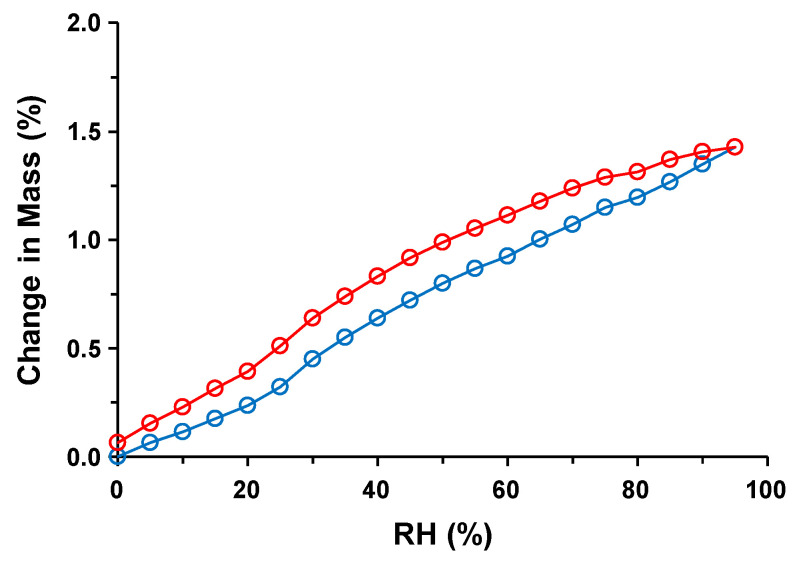
Dynamic vapor sorption (DVS) profile of Form I. Blue and red lines with points represent sorption and desorption, respectively.

Figure 6 shows the water sorption and desorption profiles of Form I. Form I was initially stored at 25 °C/0% RH in the DVS equipment, and according to the results shown in Figure 5, it should have existed as anhydrate Form I-A. Increasing RH induced water sorption in an almost linear fashion, reaching 0.32% at 25% RH. From 25% to 30% RH, the change in mass quickly increased to 0.45% and the weight increased to 95% RH: 0.6% (35% RH), 0.6% (40% RH), 0.8% (50% RH), 0.9% (60% RH), 1.1% (70% RH), 1.2% (80% RH), and 1.4% (95% RH). Despite the hysteresis loop, the desorption profile was similar to the sorption profile. At 50% RH, 0.80% sorbed water was observed, which agreed reasonably well with the weight loss of Form I up to 100 °C in the TG-DTA profile obtained under ambient conditions (Figure 3a). At 30–35% RH (Form I-B), the sorbed water comprised approximately half to two-thirds of that at 50–60% RH (Form I-C). Because the crystal form (Form I-C) did not change at 50% RH, as shown in Figure 5, the increase in weight above 60% RH reflected water sorption without a change in the water of hydration.

### 3.6. Crystal Structure Analysis

**Figure 7:**
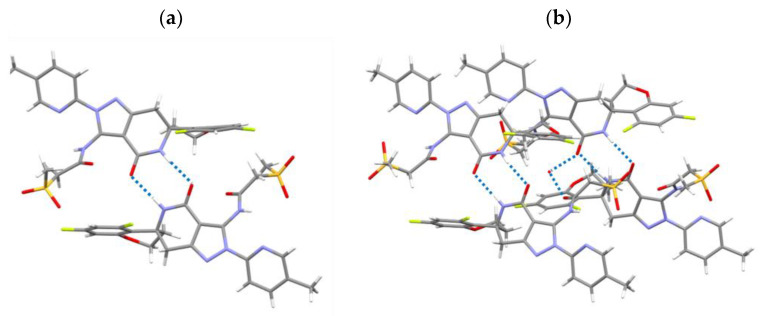
Crystal structures of (**a**) Form I-A and (**b**) Form I-C.

**Figure 8:**
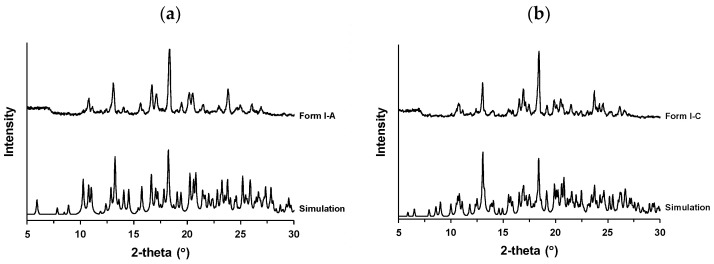
Simulated X-ray powder diffraction (XRD) profiles of (**a**) Form I-A and (**b**) Form I-C.

The crystal structures of Form I pseudo-polymorphs were subjected to single-crystal XRD analysis; however, it was not possible to obtain a single crystal of Form I-B. Figure 7a,b shows the crystal structures of Form I-A (anhydrate) and Form I-C (hydrate), respectively, and Table 2 presents their lattice parameters. The simulated XRD patterns of Forms I-A and I-C were created based on their crystal structures and reflected the typical XRD peaks observed in the experiments (Figure 8). The spaces of the two crystal forms were similar. However, the lattice spacing of Form I-A (2322.80) was slightly smaller than that of Form I-C (2349.87) owing to the lack of water for hydration. Both the crystals formed amide dimers. The two drug molecules interacted via an amide dimer in Form I-A (Figure 7a). Hydration water formed a hydrogen bond with the oxygen atom of the amide in Form I-C, and the drug-to-water ratio was 4:1 (Figure 7b). As shown in Figure 3 and Figure 6, the water of hydration constituted approximately 0.8% of the weight of Form I-C, which almost agreed with the stoichiometric value for the water of hydration of 0.25.

**Figure 9:**
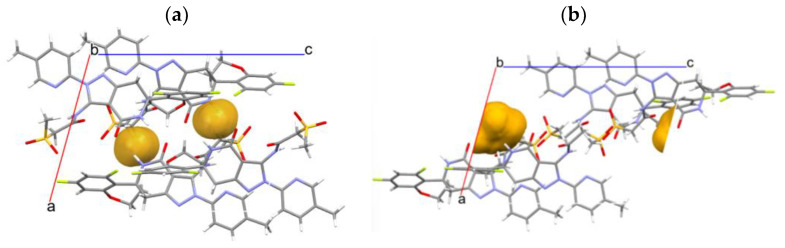
Crystal structure with void spaces of (**a**) Form I-A and (**b**) Form I-C.

The volume of void spaces is essential for the inclusion of hydrated water into a crystal lattice. The void spaces in Form I-A were calculated using the mercury voids method with a probe radius of 1.2 Å and an approximate grid spacing of 0.3 Å (Figure 9a). The obtained space available for the incorporation of the solvent into the crystal lattice was calculated to be 1.4% (32.6 Å^3^) of the total volume. The space of Form I-C when the hydrated water was excluded was 1.9% (44.8 Å^3^), obtained according to the same manner. These values are similar and suggest that the reversible hydration–dehydration of Form I does not induce a significant change in the space where water can be incorporated. Layers, voids, stoichiometric, and nonstoichiometric channel hydrates are common in pharmaceutical drugs [46]. Moreover, it has been reported the void spaces accessible to water are important for hydrate formation [47,48]. These results suggested that Form I-A retained its void spaces after dehydration to Form I-C. Consequently, the rapid rehydration of water into void spaces should be possible depending on the temperature or RH.

### 3.7. Dissolution Profiles and Stability of Form I-A and Form I-C

It is widely known that the crystal transformation between anhydrate and hydrate can cause changes in physicochemical properties, such as dissolution rate and stability, owing to different crystal lattices and/or water activities [46,49]. Investigating the effect of the transformation between Form I-A and Form I-C on physicochemical properties depending on RH is important for pharmaceutical use. However, as shown in Figure 5 and Figure 6, Form I easily and reversibly causes pseudo-polymorphic transformations depending on the RH under the experimental conditions. In our case, Form I existed as Form I-C (hydrate) because the experimental conditions were 21–25 °C and 40–60% RH. To investigate the difference in stability between the anhydrate and hydrate, Form I-C was stored at 40 °C with silica gel or at 40 °C/75%RH, according to a previous guideline [4]. It can be estimated that Form I-C maintained the hydrate at 40 °C/75%RH and transformed to the anhydrate Form I-A at 40 °C with silica gel via the XRD profiles at various temperatures and humidities (Figure 4 and Figure 5). The purity of Form I-C before storage was 99.8 ± 0.0%. The samples stored at 40 °C with silica gel or 40 °C/75%RH showed purities of 99.8 ± 0.0% and 99.7 ± 0.0%, respectively, suggesting no significant difference in stability between the anhydrate and hydrate. This result was attributed to the similar crystal lattices of Forms I-A and I-C (Figure 7 and Table 2).

**Figure 10:**
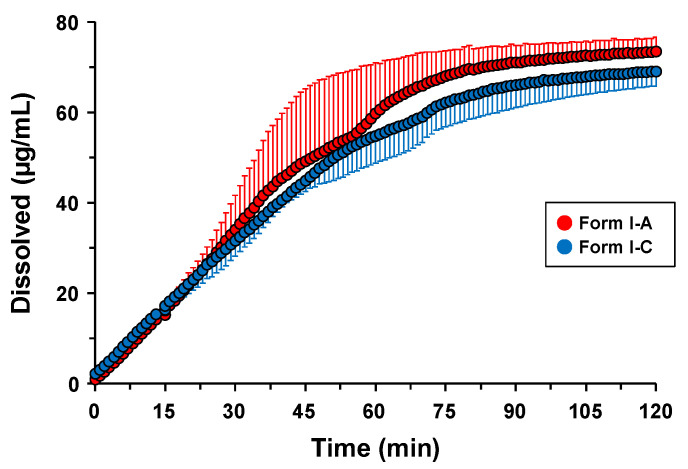
The dissolution profiles of Form I-A and Form I-C in pH 6.8 phosphate buffer at 37 °C. The error bars show the standard deviation of n = 3.

The dissolution profiles of Forms I-A and I-C were investigated. Form I-C was placed in a desiccator with silica gel and stored at 25 °C for approximately 3 h to obtain the anhydrate, Form I-A. The XRD profiles showed a quick pseudo-polymorphic transformation in response to RH, within minutes (Figure 5). Hence, Form I-C can be transformed into Form I-A after storage, which is quickly thrown into the dissolution vessel. Figure 10 shows the dissolution profiles of forms I-A and I-C. Both samples showed linear dissolution until near 45 min when the concentration reached 49.2 ± 15.9 μg/mL and 44.9 ± 2.4 μg/mL, respectively. The concentrations of Form I-A and Form I-C gently increased after 60 min and reached 73.5 ± 3.1 μg/mL and 69.0 ± 3.2 μg/mL, respectively, suggesting similar dissolution profiles between both samples. In addition to the similar crystal lattices of Form I-A and Form I-C (Figure 7 and Table 2), Form I-A rapidly transformed into Form I-C upon contact with the dissolution medium based on its rapid hydration nature (Figure 5 and Figure 6). The results of the stability and dissolution tests revealed that the transformation between the anhydrate and hydrate of S-309309 did not significantly affect the physicochemical properties, owing to the similar crystal lattices and rapid conversion to the hydrate under ambient conditions.

## 4. Conclusions

In this study, we investigated the polymorphs of S-309309 and the mechanisms by which the anhydrates and pseudo-polymorphs of the hydrates transformed into each other. Polymorphic screening identified two crystal forms, Form I and Form II. Form I was obtained using various solvents, whereas Form II could only be obtained using AcOEt. Form II was determined to be an AcOEt solvate and demonstrated thermal behavior similar to that of Form I after desolvation. The XRD profile of Form I changed reversibly with increasing temperature, and three patterns were observed. A study in which the RH was varied revealed that Form I formed anhydrate, intermediate hydrate, or hydrate according to the RH within the range of 25–60%, i.e., corresponding to ambient conditions. Single crystals of the hydrate and anhydrate were successfully prepared, and their crystal structures were investigated. The hydrate comprised a 0.25 molar ratio of water and had a crystal structure similar to that of anhydrate. The spaces in the crystal lattice enabled water sorption/desorption via an intermediate state without changing the crystal lattice. The stability and dissolution profiles of the anhydrate and the hydrate did not change significantly. These findings led to the conclusion that the pseudo-polymorphic transformation of S-309309 occurred depending on environmental conditions; however, this is not a critical issue for pharmaceutical use.

## Figures and Tables

**Table 1:** Crystal forms of S-309309 after solvent evaporation or slurry conversion.

Solvent	Solvent Evaporation	Slurry Conversion
MeOH	Form I	Form I
EtOH	Form I	Form I
IPA	Form I	Form I
AcOEt	Amorphous	Form II
Water	Form I	Form I

**Table 2:** Crystal lattice parameters of Form I-A and Form I-C.

	Form I-A (Anhydrate)	Form I-C (Hydrate)
Formula	C_23_H_21_F_2_N_5_O_5_S	C_23_H_21_F_2_N_5_O_5.25_S
Mw	517.51	521.51
Temperature (K)	213	213
Crystal system	Monoclinic	Triclinic
Space group	*P*2_1_	*P*1
a (Å)	11.68520 (10)	11.5688 (4)
b (Å)	13.35680 (10)	13.5769 (4)
c (Å)	15.4299 (2)	15.5429 (4)
α (°)	90	92.077 (2)
β (°)	105.3090 (10)	105.324 (3)
γ (°)	90	92.281 (3)
V (Å^3^)	2322.80 (4)	2349.87 (13)
Z	4	4
ρcalcg/cm^3^	1.48	1.474
μ/mm^−1^	1.797	1.789
2Θ range for data collection/°	5.902 to 153.444	5.938 to 144.38
Reflections collected	52,280	27,759
Independent reflections	17,251	8657
Goodness-of-fit on F2	1.055	1.03
Final R indexes [I ≥ 2σ (I)]	R_1_ = 0.0925, wR_2_ = 0.2629	R_1_ = 0.0765, wR_2_ = 0.2121
Final R indexes [all data]	R_1_ = 0.1037, wR_2_ = 0.2767	R_1_ = 0.0802, wR_2_ = 0.2170
Flack parameter	0.039 (8)	0.021 (7)

## Data Availability

The CIF files obtained from single-crystal X-ray diffraction are available from the Cambridge Crystallographic Data Centre: CCDC numbers 2366676 (anhydrate, Form I-A) and 2366675 (hydrate, Form I-C). The other raw data presented in this article will be made available upon request.

## Appendix A

**Table A1:** The X-ray powder diffraction (XRD) peaks (°) produced by the intact S-309309 (Form I) shown in Figure 2a.

No.	Peak Position
1	10.6
2	11.0
3	12.4
4	13.0
5	14.0
6	15.5
7	16.5
8	16.8
9	17.0
10	17.4
11	18.3
12	19.1
13	19.8
14	20.1
15	20.4
16	20.6
17	21.4
18	23.6
19	24.1
20	24.6
21	26.1

**Table A2:** The Raman peaks (cm^−1^) of the intact S-309309 (Form I) shown in Figure A1.

No.	Peak Position
1	425
2	456
3	496
4	521
5	541
6	559
7	580
8	602
9	622
10	644
11	655
12	671
13	692
14	728
15	754
16	774
17	819
18	848
19	887
20	902
21	917
22	939
23	965
24	994
25	1026
26	1071
27	1088
28	1112
29	1162
30	1194
31	1215
32	1227
33	1266
34	1320
35	1364
36	1387
37	1413
38	1448
39	1485
40	1511
41	1609
42	1650
43	1720
44	2885
45	2928
46	2947
47	2999
48	3078

**Table A3:** X-ray powder diffraction (XRD) peaks (°) of Form II shown in Figure 2b.

No.	Peak Position
1	5.6
2	6.5
3	7.4
4	9.4
5	11.2
6	12.9
7	13.7
8	15.0
9	15.8
10	16.7
11	17.2
12	17.9
13	18.0
14	18.8
15	18.9
16	20.9
17	22.2
18	23.0
19	23.9
20	24.7
21	25.1
22	26.5
23	27.7

**Table A4:** The X-ray powder diffraction (XRD) peaks (°) of Form I at the various temperatures and humidities shown in Figure 5.

No.	50 °C	25 °C/35%RH	25 °C/60%RH
1	10.8	10.7	10.7
2	11.1	11.1	11.1
3	12.4	12.4	12.4
4	13.1	13.0	13.0
5	13.5	14.0	14.0
6	14.0	15.5	15.5
7	15.6	16. 6	16.6
8	16.7	16.9	16.9
9	17.1	17.1	17.1
10	18.3	17.4	17.5
11	19.5	18.4	18.4
12	20.2	19.3	19.2
13	20.5	20.0	19.9
14	21.5	20.4	20.1
15	23.0	21.4	20.5
16	23.8	23.7	20.7
17	25.0	24.1	21.5
18	26.1	24.6	23.7
19	26.9	26.1	24.2
20	-	-	24.6
21	-	-	26.1
